# The Clinical Performance of Activa Bioactive Composite Compared to Composite in Restoring Class II Cavities in Primary Teeth: 1 Year of Split-Mouth Randomized Clinical Trial

**DOI:** 10.1055/s-0045-1811224

**Published:** 2025-08-26

**Authors:** Safaa Shihabi, Mohammed Bashier AL-Monaquel, John C. Comisi

**Affiliations:** 1 Department of Pediatric Dentistry, Faculty of Dentistry, Damascus University, Damascus, Syria; 2 Department of General Dentistry, The Dental College of Georgia at Augusta University, Augusta, Georgia, United States

**Keywords:** bioactive composite, composite, bulk-fill, class II cavities, success percentage, clinical performance, remineralization

## Abstract

**Objective:**

To evaluate the clinical performance of ACTIVA BioACTIVE compared with traditional composite in restoring class II cavities in primary teeth.

**Materials and Methods:**

In a split-mouth study design, 40 class II restorations were randomly assigned and placed in 20 children aged 6 to 9. The necessary restorations were applied according to the manufacturer's instructions. Over 12 months, two calibrated and blinded evaluators assessed the restorations at 6, 9, and 12 months using the United States Public Health Service Ryge criteria. The Wilcoxon test was used to analyze the difference in success rates between Activa bioactive composite and traditional composite after 6, 9, and 12 months, and multinomial logistic regression was also used to determine the effect of the degree and position of the caries on the success rate.

**Results:**

After 6, 9, and 12 months, the 40 restorations were evaluated. The clinical success rates for ACTIVA and composite were 95 and 85%, respectively, after 6 months, with no statistically significant difference. However, a statistically significant difference was observed after 9 months, with success rates of 90% for ACTIVA and 50% for composite ( *p* -value: 0.005). The final success rates were 85 and 45%, with no significant difference. Multiple logistic regression indicated a connection between success rates, the extent of caries, and whether the caries were located on the first or second molars. However, the restoration's location in the upper or lower jaw, as well as whether it was in the mesial or distal area, had no effect on the success rate.

**Conclusions:**

ACTIVA Bioactive composite may serve as a viable option for restoring primary teeth; however, further studies with longer follow-up periods and larger sample sizes are necessary.

**Clinical Significance:**

Using ion-releasing materials can enhance clinical restorative success as documented in this clinical study.

## Introduction

Dental caries remains the most common chronic disease, negatively impacting the oral health of both adults and children, 1 and consequently affecting their quality of life. 2 Therefore, treating dental caries is essential. Additionally, the current trend in dentistry emphasizes aesthetic concerns, even in pediatric dentistry; however, the ideal aesthetic restoration material does not yet exist. 3

Achieving ideal composite restorations presents a challenge in adults and is even more difficult in pediatric dentistry, particularly with uncooperative patients. These complexities stem from precise application, multiple steps, inadequate light energy, polymerization shrinkage, degradation of the interfacial bond, and microleakage. As a result, these factors can lead to recurrent caries and restoration failures, necessitating frequent replacements. 4

Many other restorative materials have been introduced to the market to prevent complications associated with the placement of composite materials. One such material is resin-modified glass ionomer (RMGI), 3 which can release ions that support remineralization. Compared with glass ionomer cement (GIC), RMGI offers enhanced adhesion and sealing properties while also addressing patients' aesthetic needs. However, its abrasion resistance continues to be a topic of debate.

Recently, a bioactive composite has been developed as a restorative material that combines the desirable properties of composites and RMGI. It releases calcium, phosphate, and fluoride ions in restored teeth as needed by the oral environment, raising pH levels and restoring the balance in the oral cavity toward remineralization. 5 These ions can seal the gaps caused by polymerization shrinkage, reducing the rates of microleakage, sensitivity, and secondary caries, which may be particularly beneficial for patients at high risk of dental caries. 6 7 8 9 10

ACTIVA Bioactive is a dual-cure ionic restorative material with a bioactive resin matrix that mimics the physical and mechanical properties of natural teeth. It can release and recharge calcium, phosphate, and fluoride. Additionally, it is a dynamic and “smart” material that responds to pH changes in the oral cavity. 11 Moreover, it is designed as a bulk-fill material, allowing clinicians to place 4 to 5 mm increments, streamlining the procedure, reducing chair time, and positively influencing patient behavior. 12

To the best of our knowledge, the number of studies on bioactive composites in primary teeth remains limited. Therefore, this study compares the clinical performance of bioactive composites with traditional composites in restoring class II cavities in primary teeth.

## Materials and Methods

### Ethical Approval, Trial Registration, and Informed Consent

The ethical approval for this study was obtained from the Damascus University Ethics Committee (decision number 2327, 4/18/2022). The trial was registered with ISRCTN under the registration number ISRCTN98762227 at https://www.isrctn.com/ISRCTN98762227 .

All the volunteers were residents of Dar al-Aman orphanage in Damascus, Syria. The mothers, guardians, or caregivers signed the consent form and were informed about all the steps, duration, benefits, and possible complications.

### Trial Design and Blinding

The study was designed as a double-blind randomized clinical trial utilizing the split-mouth technique. Neither the patients nor the evaluators were aware of the materials used in this study, which was conducted in accordance with the guidelines of the CONSORT statement.

### Sample Size Calculation

Using G*power (version 3.1.9.7) at a 95% confidence level, with a significance level of 5%, and based on similar previous studies, the sample size was calculated to be 40 restorations for 20 patients aged 6 to 9, depending on the standard deviation.

### Randomization

After examining the patients and to avoid selection bias, the study achieved randomization through a straightforward method as described below:

Each patient in this study will receive two distinct restorative materials: bioactive composite (ACTIVA KIDS BioACTIVE, Pulpdent) on one side and composite (Tetric n-ceram, Ivoclar Vivadent) on the other, with randomization occurring in two stages. The components of the two materials are detailed in Table 1 .

**Table 1:** The composition or restoration materials used in the study

Materials	Composition	Manufacturers
ACTIVA KIDS	Blend of diurethane and other methacrylates with modified polyacrylic acid, silica, amorphous sodium fluoride	PulpDent Watertown, Massachusetts 02472, United States
Tetric n-ceram	Monomer matrix (Bis-MMA, UDMA, Bis-EMA), fillers contain barium glass, ytterbium trifluoride mixed oxide and copolymers.	Ivoclar Vivadent, Liechtenstein

The patient was asked to roll a die twice: first to select a side and a second time to choose the restorative material as follows:

With the first roll of the dice, the numbers 1, 3, 5 denote the right side of the jaw, while the numbers 2, 4, and 6 denote the left side. In the second roll of the dice, 1, 3, and 5 indicate Activa bioactive, whereas the numbers 2, 4, and 6 indicate composite.

### Patient Inclusion Criteria

After examining 100 children living at the Dar al-Aman orphanage, only 20 were included in this study based on inclusion criteria that required cooperative, healthy patients (A1) according to the American Society of Anesthesiologists, aged between 6 and 9, and having two carious lesions, one on each side of the mouth. 13

### Teeth Inclusion Criteria

**Fig. 1:**
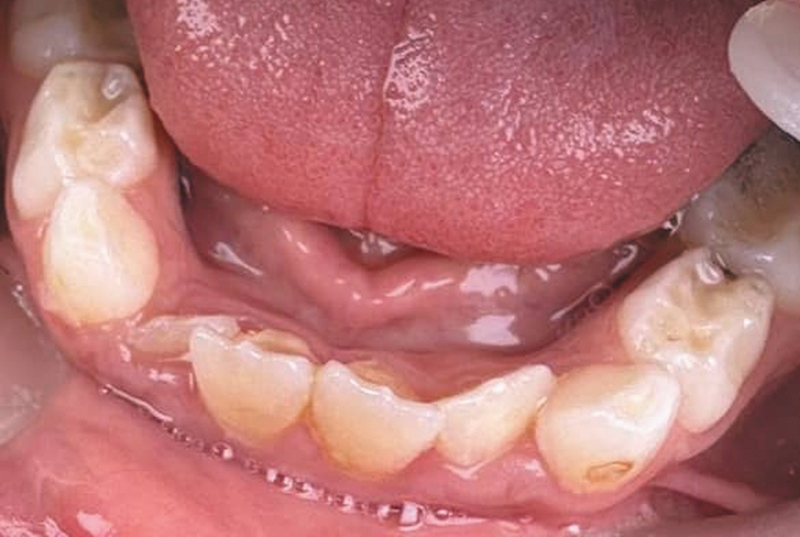
Caries on 7,4 and 8,4 before cavity preparation and isolation.

The selected teeth were first or second primary molars from both sides and both jaws (upper and lower) that exhibited interproximal dental caries classified as fourth degree (underlying dark shadow from dentin) or fifth degree (distinct cavity with visible dentin) according to the International Caries Detection and Assessment System (ICDAS). 14 The teeth must be vital (with no mobility or fistula), restorable, and symptom-free, showing no signs of pulpal inflammation or pathological lesions ( Fig. 1 ). Any patients who did not meet these criteria were excluded from the study. 15

### Clinical Procedure

During the first session, all patients received education on brushing their teeth and were given oral care instructions.

During the second session, topical anesthesia was applied, followed by infiltrative local anesthesia for the cavities in the upper maxilla and an inferior alveolar nerve block for the mandibular cavities.

**Fig. 2:**
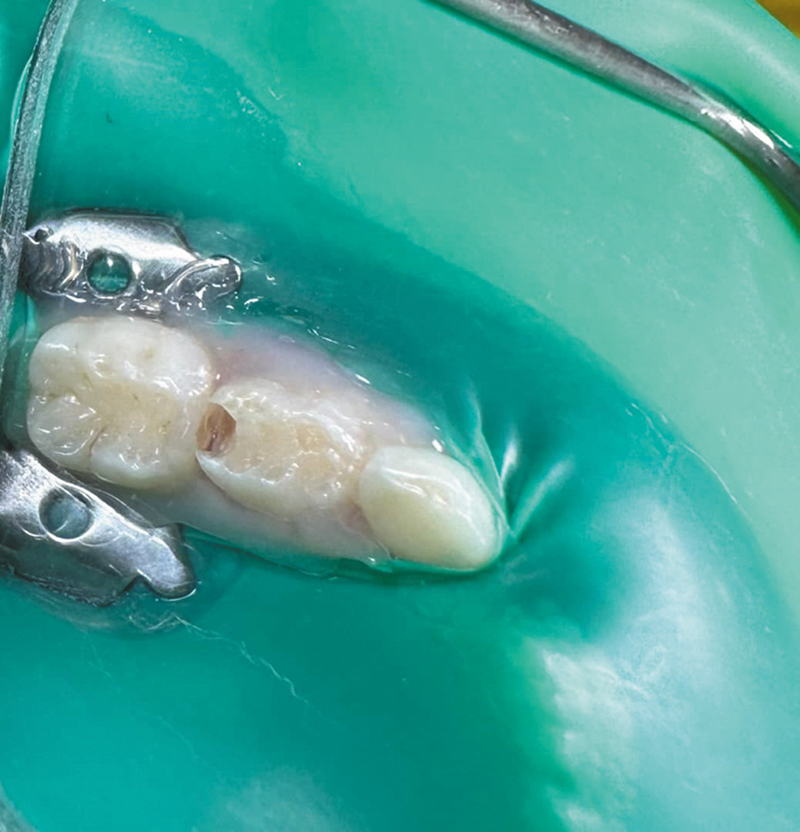
The isolation and preparation of the cavity.

After applying the rubber dam ( Fig. 2 ), cavities were prepared using a straight fissure diamond (Zeffiro; Italy) with high-speed rotary handpieces (NSK S-Max M800L) equipped with air–water cooling. The smooth, infected dentin was removed with hand excavators or a low-speed handpiece (NSK S-Max M25L) until hard dentin was reached, keeping the following points in mind. 16

The bucco-lingual dimension of each proximal cavity preparation was located in the middle third between the cusps; the gingival wall was directly below the proximal contact points, and the unsupported enamel needed to be removed ( Fig. 2 ). The prepared walls were finished using an extra-fine diamond bur (Zeffiro; Italy) to round all the line angles, and the entire cavity preparation was rinsed for 10 seconds and gently air-dried.

After preparing the cavity, a wooden wedge and matrix band were placed interproximally to receive the restorative material, according to the manufacturer's instructions outlined below:

For ACTIVA Bioactive composite, selective etching was performed for 30 seconds on enamel and 15 seconds on dentin. The etching agent was rinsed off and gently dried to prepare for the bonding agent, a fifth-generation product (Tetric n-Bond, Ivoclar Vivadent). This bonding agent was applied, gently air-dried, and then light-cured for 20 seconds. A mix-tip cannula was placed at the cavity floor and kept submerged in the material to prevent air bubbles. Activa was applied using a bulk-fill technique in 4 mm increments. Since the material is dual-cure, the initial cure is achieved via light curing at 500 mW/cm ^2^ for 20 seconds. This initiates the dual curing of the material, which will completely set in the oral cavity within 3 minutes. 11

For traditional composites, cavity etching and bonding were conducted in the same manner as in the Activa group after washing and drying. The composite was then applied incrementally in layers, each up to 2 mm thick. After each layer, a light-curing device was used to cure the material at 500 mW/cm ^2^ for 20 seconds. 17

**Fig. 3:**
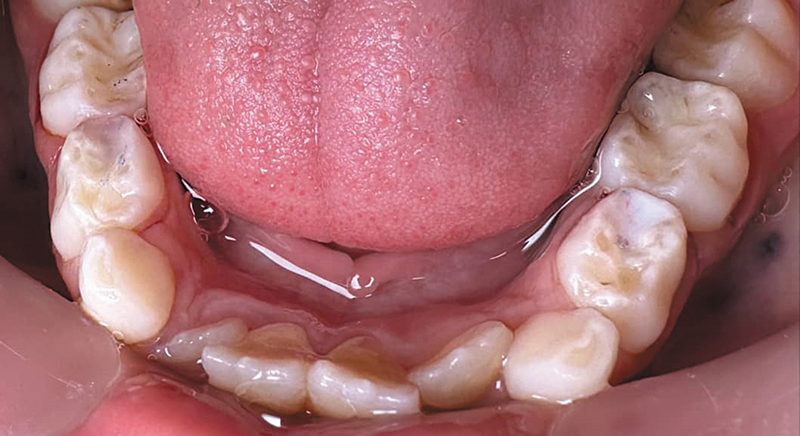
The restorations after finishing, 7,4 restored with Activa, 8,4 restored with composite.

Finally, the occlusion of both restorations was verified with articulating paper, and the procedure was completed using superfine diamond burs, polishing strips, and rubber cups ( Fig. 3 ).

All clinical procedures were performed by a single clinician, who is a pediatric dentist.

### Clinical Evaluation

The restorations were clinically evaluated at baseline, 6, 9, and 12 months using the United States Public Health Service (USPHS) clinical rating system. This system assesses the following criteria: anatomical form, marginal integrity, marginal discoloration, fracture, color stability, recurrent caries, and surface texture. Each criterion is assigned one of three values: A indicates a successful restoration, B signifies an acceptable restoration, and C denotes a failure. 18

At each clinical evaluation appointment, the teeth were professionally cleaned with a slow-speed brush to remove dental plaque and food debris. Subsequently, two blinded, calibrated pediatric dentists, trained to utilize the USPHS system by evaluating 50 previously placed restorations, examined the restoration with a right-angled dental explorer and loupes.

### Statistical Analysis

The results were analyzed using SPSS 23.0 software. A *p* -value of less than 0.05 was considered statistically significant for all tests.

The Kolmogorov–Smirnov test indicated that the data were not normally distributed. The difference between the two groups (ACTIVA and composite) at various time points was analyzed using the Wilcoxon test. Intra-examiner reliability was assessed using the Kappa test.

Multiple logistic regression was used to evaluate the relationship between success percentage and several variables: the severity of caries, the location of the restoration in the upper or lower jaw, whether it involved the first or second molar, and whether the restoration was mesial or distal.

## Results

Twenty boys, with an average age of 6.6 years, received 40 restorations. Table 2 presents the descriptive statistics of the restorations included in the study.

**Table 2:** The descriptive statistics of the restoration distribution in the study sample

	Upper or lower jaw		First or second molar		Mesial or distal restorations		Caries degree according to ICDAS	
Percentage %	Upper jaw	Lower jaw	First molar	Second molar	Mesial	Distal	(4) Degree	(5) Degree
	70%	30%	50%	50%	55%	45%	62.5%	37.5%

Abbreviation: ICDAS, International Caries Detection and Assessment System.

The Kappa test demonstrated a strong inter-examiner agreement ( *p* -value = 0.000), as shown in Table 3 .

**Table 3:** Kappa test for inter-examiner reliability

	Samples	Kappa	*T* -value	*p* -Value
Success rate indexes	40 restorations	1.0	6.325	0.000

**Fig. 4:**
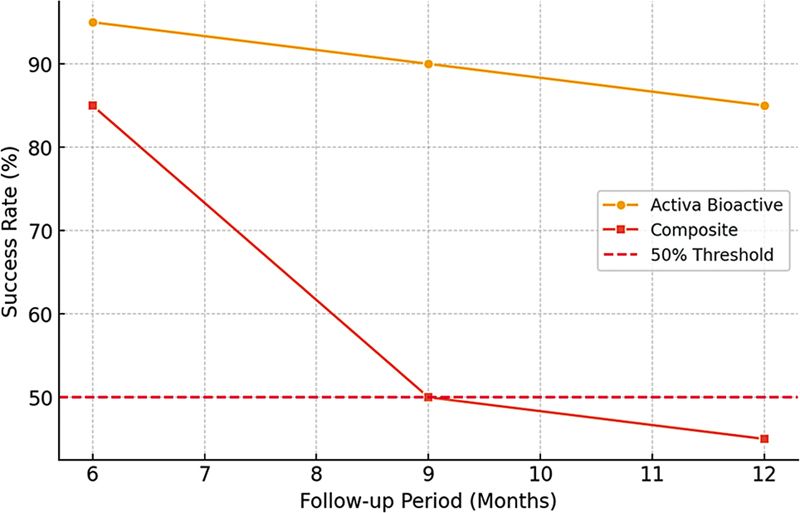
Comparison of success rates between Activa and composite.

As shown in Table 4 and Fig. 4 , 85% of Activa restorations were successful after 12 months, as compared with 45% for composites.

**Table 4:** Descriptive statistics of the success percentage of restoration materials within the follow-up period

	ACTIVA			Composite		
	Success	Acceptable	Failure	Success	Acceptable	Failure
6 months	95%	5%	0%	85%	15%	0%
9 months	90%	10%	0%	50%	50%	0%
12 months	85%	5%	10%	45%	55%	5%

The Wilcoxon test showed no significant differences in clinical performance between Activa and composite at the 6- and 12-month follow-ups ( *p* -values of 0.317 and 0.109, respectively). However, a significant difference was observed at the 9-month mark ( *p* -value of 0.005), indicating that Activa outperformed the composite ( Table 5 ). Multiple logistic regression is shown in Table 6 .

**Table 5:** Comparison of success rate between restorative materials within the follow-up period

Follow-up (months)	Activa success (%)	Composite success (%)	*p* -Value
6	95	85	0.317
9	90	50	0.005
12	85	45	0.109

**Table 6:** Multiple logistic regression between success rate and many variables

	*B*	Std. error	Wald	df	Sig	Exp ( *B* )	CI for exp ( *B* )	
							Lower bound	Higher bound
Success rate	0.927	1.030	3.031	1	0.368			
Caries degree (4/5)	1.856	0.857	0.140	1	0.030	6.398	0.179	12.608
Mesial/distal	0.167	0.885	0.007	1	0.850	1.182	0.101	8.237
Upper/lower	0.330	0.922	0.042	1	0.720	1.391	0.068	8.830
First/second	−2.256	0.977	0.862	1	0.021	0.105	0.026	3.669

Abbreviation: CI, confidence interval.

The USPHS clinical criteria ratings at the 1-year follow-up can be found in Table 7 .

**Table 7:** USPHS clinical criteria ratings at 1 year of follow-up

Restoration materials	Activa			Composite		
	A	B	C	A	B	C
Anatomic form	85%	5%	10%	45%	55%	0%
Marginal adaptation	85%	5%	10%	55%	45%	0%
Marginal discoloration	100%	0%	0%	90%	10%	0%
Fracture	90%	10%	0%	100%	0%	0%
Color match	100%	0%	0%	90%	10%	0%
Secondary caries	95%	5%		95%	5%	
Surface texture	100%	0%		90%	10%	

Note: A: successful restoration, B: acceptable restoration, C: failure.

## Discussion

Dental caries is the most common chronic disease and the leading cause of tooth loss, especially among children. 19 Therefore, restorative treatments for these caries are essential for maintaining both aesthetic and functional aspects, thus enhancing quality of life. 20 Recently, the aesthetic appearance of children has gained increasing significance, leading to a decline in the use of stainless-steel crowns and amalgam restorations in favor of composite and other aesthetic restoration materials. 21

Achieving an ideal composite restoration in primary teeth is particularly challenging due to the sensitivity during application, which requires strict isolation and extended working time. These conditions are difficult to maintain with children. 22 Moreover, polymerization shrinkage can lead to marginal microleakage, and insufficient light energy from curing lights can contribute to sensitivity, recurrent caries, and ultimately restoration failure, presenting significant challenges to its use. 23 Consequently, several alternatives, such as RMGI, have been introduced to the market; this material meets aesthetic requirements and releases fluoride, making it a viable alternative to composite and GIC, especially for high-risk patients. However, its physical and mechanical properties remain debatable. 17

Bioactive materials have gained popularity in pediatric dentistry, significantly advancing the field. Their appeal stems from their ability to promote remineralization in the oral cavity, a key objective of restorative materials. 24

This study aimed to evaluate the clinical performance of the ACTIVA Bioactive composite, applied using the bulk-fill technique to restore Class II cavities, and to compare it with the composite over 1 year of follow-up.

Class II cavities in primary teeth were selected due to the complexity of their restoration. The interproximal contact between teeth is surface-to-surface, which makes achieving an ideal restoration challenging. 25

The study was conducted at Dar al-Aman orphanage because the residents have similar oral health and diets. This creates uniformity in the quantity and quality of meals and snacks, making the study as controlled as possible.

This clinical study utilized a split-mouth controlled trial, where each restoration served as a control for the other within the same patient. This method minimized the sample size and the likelihood of false positives. 26

An analysis using multiple logistic regression to examine the success rate (dependent variable) in relation to various independent factors over 9 months showed that initial higher levels of caries negatively affect the success rate, as seen in Table 6 . Interestingly, the presence of caries on the second molars increases the success rate, as indicated in Table 6 . Conversely, no significant impact on the success rate was found when caries were present in the upper or lower jaw, or in the mesial or distal regions of the teeth.

Furthermore, the success rate improved when our restoration included second molars, probably because anatomical and procedural factors, such as their larger size than first molars, make the materials easier to adapt the restoration.

The clinical success rate of ACTIVA was 85% after 12 months, compared with 45% for composite restorations for anatomic form, and 85 and 55% for marginal adaptation, ACTIVA versus composite ( Table 7 ). Interestingly, there was no statistically significant difference between the two; however, a statistically significant difference was observed after 9 months of follow-up, possibly due to the high sensitivity of the application technique for composite restoration. Compared with the success percentage after 6 months, we can see that the functional properties of both materials changed over time. This result aligns with Lardani et al. 27 When we examine the data plotted on a graph, as shown in Fig. 4 , we can note the difference in success rates between Activa and the composite. The success rate falls below the 50% level at 12 months. Although this may not have statistical significance compared with the Activa success rate, it does indicate a substantial decline in success.

This substantial decline suggests that the materials used and how they were applied (incremental and bulk-fill) can significantly affect outcomes.

It has been suggested that the overall longevity of incrementally placed composites differs from that of bulk-fill materials. 28 29 Incremental placement can decrease stress caused by polymerization shrinkage by limiting the volume cured at each step; however, this does not guarantee the elimination of marginal gaps or failures over time. Additionally, operator error remains a significant factor that can negatively impact long-term outcomes. Bulk-fill composite materials allow for larger placements, are designed to help reduce shrinkage stress, and improve depth of cure. 30 These materials may also lower the chances of void formation during placement since a single layer can often be used, reducing procedure time and offering significant benefits for restoring pediatric dentition. 31 And if the material also has the potential benefit of resisting secondary caries through ion release, it can help improve the longevity of the restoration, keeping it above the 50% threshold as illustrated in Fig. 4 .

The limitations of the study include a small sample size and it being conducted at a single location. The final 12-month results do not show a significant difference in the materials, unlike the 9-month results. However, Fig. 4 graphically shows the difference in success rates for each material. Although the 12-month data do not indicate a statistically significant difference, it suggests that one material failed faster than the other over time. Additionally, despite lacking statistical significance at 12 months, the overall decrease in success rate between the materials is noteworthy. Longer term studies are needed to further investigate these materials.

## Conclusion

Despite this study's limitations, we can conclude that ACTIVA Bioactive composite may be the preferred material for restoring class II cavities in primary teeth due to high success rate, facilities in application, and bulk fill efficiency, which is considered effective in reducing chair time for children and thereby reflecting positively on children's cooperation. However, further clinical studies with large sample size and extended follow-up periods with other bioactive materials are necessary.
